# Differentiation of Pre- and Postganglionic Nerve Injury Using MRI of the Spinal Cord

**DOI:** 10.1371/journal.pone.0168807

**Published:** 2016-12-30

**Authors:** Amar Karalija, Liudmila N. Novikova, Greger Orädd, Mikael Wiberg, Lev N. Novikov

**Affiliations:** 1 Department of Integrative Medical Biology, Section of Anatomy, Umeå University, Umeå, Sweden; 2 Department of Surgical and Perioperative Science, Section of Hand and Plastic Surgery, Umeå University, Umeå, Sweden; 3 Department of Integrative Medical Biology, Section of Physiology, Umeå University, Umeå, Sweden; 4 Umeå Centre for Comparative Biology, Umeå University, Umeå, Sweden; Szegedi Tudomanyegyetem, HUNGARY

## Abstract

Brachial plexus injury (BPI) is a devastating type of nerve injury, potentially causing loss of motor and sensory function. Principally, BPI is either categorized as preganglionic or postganglionic, with the early establishment of injury level being crucial for choosing the correct treatment strategy. Despite diagnostic advances, the need for a reliable, non-invasive method for establishing the injury level remains. We studied the usefulness of *in vivo* magnetic resonance imaging (MRI) of the spinal cord for determination of injury level. The findings were related to neuronal and glial changes. Rats underwent unilateral L4 & L5 ventral roots avulsion or sciatic nerve axotomy. The injuries served as models for pre- and postganglionic BPI, respectively. MRI of the L4/L5 spinal cord segments 4 weeks after avulsion showed ventral horn (VH) shrinkage on the injured side compared to the uninjured side. Axotomy induced no change in the VH size on MRI. Following avulsion, histological sections of L4/L5 revealed shrinkage in the VH grey matter area occupied by NeuN-positive neurons, loss of microtubular-associated protein-2 positive dendritic branches (MAP2), pan-neurofilament positive axons (PanNF), synaptophysin-positive synapses (SYN) and increase in immunoreactivity for the microglial OX42 and astroglial GFAP markers. Axotomy induced no changes in NeuN-reactivity, modest decrease of MAP2 immunoreactivity, no changes in SYN and PanNF labelling, and a modest increase in OX42 and SYN labeling. Histological and radiological findings were congruent when assessing changes after axotomy, while MRI somewhat underestimated the shrinkage. This study indicates a potential diagnostic value of structural spinal cord MRI following BPI.

## Introduction

Traumatic brachial plexus injury (BPI) is a severe form of nerve injury, potentially causing a devastating loss of sensory and motor function. Principally, BPI may either occur as a complication to childbirth [1], or as a post-traumatic injury in adults, most often secondary to traffic accidents. [2] The current treatment approach is largely dependent on the level of injury, with a distinction being made between the preganglionic nerve injury to the spinal roots, and the postganglionic nerve injury to the spinal nerves. [3] In the case of the latter injury type, function may either spontaneously return with time or, when loss of nerve continuity occurs, the nerve can be repaired using an autologous nerve transplant, which is normally harvested from the patients leg. Preganglionic avulsion injury is a severe form of preganglionic nerve injury that causes a disconnection between the central and peripheral nervous systems. Avulsion injury, considered the most severe type of preganglionic injury, is associated with a marked and progressive death of motoneurons [4, 5] with a very poor prognosis for functional recovery. Currently, there is no generally approved, efficient treatment for the avulsion injury.

In order to provide the appropriate treatment strategies for brachial plexus injuries, the level of injury needs to be established. A range of different modalities have been used over the years, with current diagnostics relying on a combination of clinical testing, electrodiagnostic tests, CT myelography and MRI. [6] No diagnostic modality alone seems to suffice, and several tests may have to be employed. In some instances surgical exploration is required for the final determination of the injury level. Therefore, there is a persisting need for a non-invasive, reliable and validated method of accurately establishing the level of brachial plexus injury. Early repair of nerve injury is associated with an increased survival of neurons [7–9] and repair within 2 months after nerve axotomy is associated with an improved functional outcome. [10] Therefore a reliable diagnostic tool capable of early detection and establishment of the level of injury is desirable for planning and early repair of the plexus injury.

Compared with axotomy of the peripheral nerve, ventral root avulsion, due to its severity, causes more extensive changes in the corresponding spinal cord segments. From previous studies it is known that ventral root avulsion causes an extensive death of motoneurons after 4 weeks. [5, 11] Furthermore, ventral root avulsion has been shown to induce microglial and astroglial reactivity in the ventral horn as well as an extensive loss of synaptic boutons. [12, 13] Conversely, peripheral nerve axotomy does not induce a significant loss of motoneurons at 4 weeks after injury, with the death of motoneurons being observable first after 6 weeks [14] and reaching approximately 30% by 16 weeks. [9, 15] Comparing dorsal root avulsion and corresponding rhizotomy, avulsion has been shown to induce a significantly greater loss of both neurons and non-neuronal cells in the dorsal columns and dorsal horn of the spinal cord. [16] Therefore, we postulate that other aspects of the spinal cord grey matter may be differently affected by the two injury types, such as the dendritic and axonal systems surrounding the cells.

In this study, we used lumbar L4-L5 ventral root avulsion and sciatic nerve transection in rats as models for study of preganglionic and postganglionic injury in humans, respectively. We developed an MRI protocol for *in vivo* investigation of possible morphological grey matter changes secondary to preganglionic avulsion and postganglionic injury in rats. Our aim was to relate possible changes observed using MRI to corresponding histological preparations of the spinal cord. In this manner the histopathological basis for the MRI findings might therefore be enabling validation of the method. Our aim was also to establish whether structural MRI could be used to differentiate these two fundamentally different injury types.

## Materials and Methods

### Experimental model and ethics statement

The experiments were performed on adult (10–12 weeks, n = 9) female Sprague-Dawley rats (Taconic Europe A/S, Denmark). The animal care and experimental procedures were carried out in full compliance with Directive 2010/63/EU of the European Parliament and of the Council on the protection of animals used for scientific purposes. The study was also approved by the Northern Swedish Committee for Ethics in Animal Experiments (No. A36-12 and A186-12). All surgical procedures were performed under general anaesthesia using a mixture of ketamine (Ketalar^®^, Parke-Davis; 100mg/kg i.v.) and xylazine (Rompun^®^, Bayer; 10mg/kg i.v.). After surgery, the rats were given benzylpenicillin (Boehringer Ingelheim; 60mg, i.m.). Each animal was housed alone in a cage after surgery and exposed to 12-hour light/dark cycles, with ad lib access to food and water. After the conclusion of the experiments, the experimental animals were deeply anaesthetised by administration of an intraperitoneal overdose of pentobarbital (240 mg/kg, Apoteksbolaget, Sverige).

### Sciatic nerve axotomy

The experimental animals (n = 5) underwent a unilateral left side transection of the sciatic nerve, performed at the upper border of the quadratus femoris. To prevent spontaneous regeneration of the nerve, the proximal as well as the distal stump were covered with a custom made blind-ending polyethylene cap that was sutured to the epineurium using Ethilon^™^ 9–0 sutures.

### L4 and L5 ventral root avulsion

After performing a lumbar laminectomy, the L4 and L5 ventral roots were identified (n = 4). Following transection of the roots with a pair of Vannas spring scissors, the proximal stump of each transected nerve ventral root was grasped using jeweller’s forceps. The roots were slowly pulled in the caudal direction, tangentially to the spinal cord, until the root was ruptured and came out in its entire length. Post mortem, the spinal cord was inspected and the L4 and L5 ventral roots found to be avulsed from their respective segment.

### Magnetic resonance imaging (MRI) acquisition & subsequent quantification of the ventral horn area on MRI

All scans were performed on a 9.4 T Bruker BioSpec 94/20 USR system connected to a mouse heart array coil combined with a 87 mm QUAD resonator coil and running ParaVision^®^ software (Bruker BioSpin Group, Bruker Corporations, Germany). The experimental animals underwent anaesthesia using isoflurane (Attane vet^®^, 1000 mg/g, Oiramal Healthcare, UK). The isoflurane was continuously administered through a custom-made breathing mask connected to the animal bed. Respiration and temperature were monitored using a respiration pillow and a rectal probe respectively (SA Instruments Inc., Stony Brook, USA).

Firstly, a series of orientational pilot scans were performed in order to establish the position of the animal and identify anatomical landmarks relevant for the planning of the subsequent scans. A coronal scan of the thoracic spine was used to establish the position of the 13^th^ rib, with the caudal tip of the first caudally situated DRG, DRG 13, corresponding to the L4 spinal ventral root entry zone. The obtained images were then used to position a total of 8 image slabs oriented transversely, and spanning the L4 and L5 segments. Account was taken to the somewhat curved shape of the spinal cord, with all the slabs being placed at 90° in relation to the cord. The data was acquired with a T2-weighted TurboRARE sequence (TR 2000.0 ms, TE 11.0 ms, field of view 20x20 mm, matrix 256x256, slice thickness 2 mm, 4 averages). Respiration gating was employed, giving an approximate acquisition time of 35 minutes.

**Fig 1:**
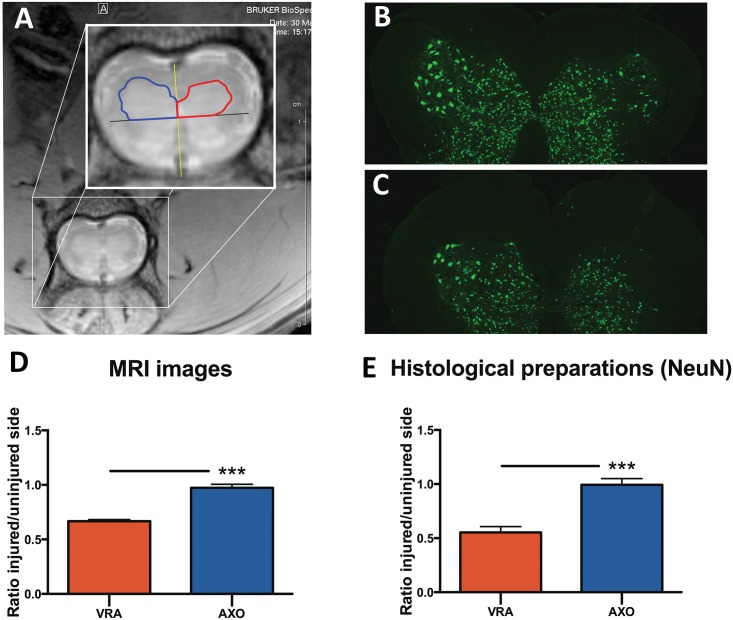
Assessment of ventral horn size on MRI & histological measurements of the ventral horn neuron pool size. Axial image of the L4/L5 spinal cord segment following ventral root avulsion, with the spinal cord divided in a right and a left side (yellow line), and the ventral horn separated from the dorsal horn (black line). The ventral horn area is outlined on the injured (red line) and uninjured side (blue) (A). The equivalent measurement was performed in histological preparations of the spinal cord sections stained with NeuN after axotomy (B) and ventral root avulsion (C). The histogram shows the relative area ratios obtained by measurements of the ventral horn area in MRI images (D) and histological preparations (E) after ventral root avulsion (VRA) and axotomy (AXO). Error bars show S.E.M. P<0.001 is indicated by ***.

The obtained images were exported to DICOM format from ParaVision^**®**^ and converted to high quality TIFF format pictures. The pictures were assessed using Image-Pro Plus software (Media Cybernetics, Inc., USA). Firstly, a line was drawn running through the central canal of the spinal cord and the anterior spinal artery, thereby dividing the spinal cord in a left and a right side. Another line was then drawn, running in a lateral direction from the central canal and being positioned at a 90° angle to the first line (Fig 1A). These two reference lines together produced the anterior-posterior and medial-lateral border of the left and right ventral horn. Using the lines as a reference, the ventral horns on both sides were outlined, and the area on each side was measured. Finally, a ratio between the area of the injured and uninjured side was calculated.

### Tissue processing

After the conclusion of the experiments, i.e. after 4 weeks, the experimental animals were deeply anaesthetised by administration of an intraperitoneal overdose of pentobarbital (240 mg/kg, Apoteksbolaget, Sverige). The animals were transcardially perfused using Tyrode´s solution followed by 4% paraformaldehyde (PFA) dissolved in 0.1 M phosphate buffer (pH 7.4). Following perfusion the spinal cord segments L4 and L5 were harvested and post-fixed in PFA, after which the tissue underwent cryoprotection in 10% and 20% sucrose for 3 days, finally to be frozen in liquid isopentane. The tissue was cut in 16 μm-thick serial sections on a cryomicrotome (Leica Instruments), thaw-mounted onto SuperFrost^®^ Plus slides, dried overnight at room temperature and stored at -85°C before being further processed.

### Immunohistochemistry

The serial sections underwent immunohistochemical processing for the identification of neuronal and glial cell markers. Following blocking with normal serum, the following primary antibodies were applied: mouse anti-neuronal-nuclei antibody (NeuN; 1:200, Chemicon), mouse anti-microtubule-associated protein-2 (MAP2; 1:200, Chemicon), rabbit anti-synaptophysin (SYN; 1:500, Dako), rabbit anti-glial fibrillary acidic protein (GFAP; 1:1000, Dako), monoclonal antibodies reacting with C3bi complement receptors (OX42; 1:200, Serotec) and a cocktail of monoclonal antibodies reacting with 68 kDa, 160 kDa and 200 kDa neurofilament proteins (NF; 1:200; Zymed Laboratories). The primary antibodies were applied at room temperature for 2 hours. Following rinsing in PBS, secondary goat anti-mouse and goat anti-rabbit antibodies Alexa Fluor^®^ 488 and Alexa Fluor^®^ 568 (1:300; Molecular Probes, Invitrogen) were applied for 1 h in darkness and at room temperature. All the slides were coverslipped with ProLong mounting media containing DAPI (Invitrogen). The staining specificity was tested by the omission of primary antibodies.

### Morphological analysis of the ventral horn on histological preparations

The area of the ventral horn was analysed on NeuN-immunostained preparations of the L4-L5 spinal cord segments, containing NeuN positive neurons. The ventral horn and the adjacent lateral and anterior funiculus were photographed at 4x magnification using a Nikon DS-U2 digital camera. The images were captured randomly, excluding damaged sections and sections containing large blood vessels and artifacts. The images were assessed using the same software and the identical standardized protocol that was employed when assessing the MRI pictures, as described previously (please see *Magnetic resonance imaging (MRI) acquisition & subsequent quantification of the ventral horn area on MRI*). The ratio between the area of the injured and healthy side was calculated correspondingly.

When assessing the neurofibrillary, synaptic and glial cell markers, a ventral region of the L4-L5 ventral horn on the border with the white matter was chosen. Images were captured randomly at 40x magnification, with the exclusion of sections where the area of interest was damaged, or when blood vessels or artifacts interfered with the picture quality. Six images per animal were captured with three images representing the injured side and three images representing the contralateral side. The area occupied by the immunostained profiles was calculated using Image-Pro Plus software (Media Cybernetics, Inc., USA), employing a standardized protocol.

### Statistical analyses

Statistical differences between the experimental groups were established by performing an unpaired t-test (Prism^®^, GraphPad Software, Inc; San Diego, CA). The statistical significance was set at *p<0.05, **p<0.01, ***p<0.001.

## Results

### Quantification of the ventral horn area using MRI

In animals undergoing sciatic nerve axotomy, no statistically significant difference in the ventral horn size was found between the injured side and the contralateral side (Fig 1D) after 4 weeks. In animals undergoing ventral root avulsion, a statistically significant difference was found between the injured and the non-injured side (p<0.001), with a 34±0.7% shrinkage of the ventral horn area on the injured side compared to the healthy side (Fig 1D). A statistically significant difference in the ventral horn area ratio was seen between animals subjected to axotomy compared to animals undergoing avulsion (p<0.001), indicating that the two different types of injuries could be distinguished from each other (Fig 1D).

### The morphology of the ventral horn on histological preparations

#### Changes in ventral horn area in NeuN-immunostained sections

The changes in the area of the ventral horn occupied by neurons was assessed by determining the size of the area containing NeuN-positive neurons. In analogy with the method employed when calculating the ventral horn size in MRI pictures, a relative ratio of shrinkage was calculated for every animal. In animals undergoing axotomy, no statistically significant shrinkage in the size of the neuron pool was observed 4 weeks after injury (Fig 1B and 1E). Animals subjected to ventral root avulsion exhibited a shrinkage of 44%±1.5% on the injured side compared to the non-injured side (Fig 1C and 1E). A strong statistical difference regarding the size ratio of the neuron pool was found between animals that underwent axotomy compared to ventral root avulsion (p<0.001), with the latter exhibiting a much smaller area ratio (Fig 1E).

#### The density of dendrites, axonal terminals and synaptic boutons

Changes induced by axotomy and ventral root avulsion on dendrites, synaptic boutons and axons belonging to the neurons of the ventral horn were investigated by staining with MAP2, synaptophysin and pan-neurofilament respectively.

**Fig 2:**
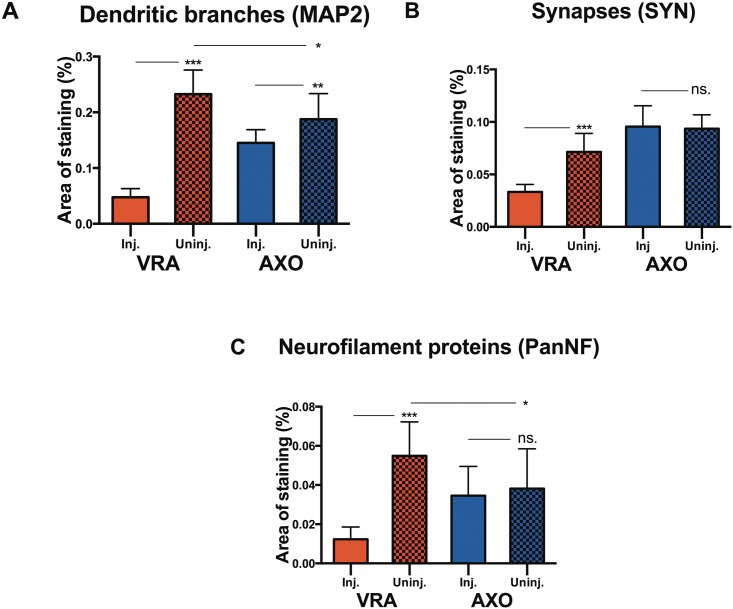
Quantification of dendrites, synapses & axons. Histogram showing the relative tissue area occupied by MAP2-positive dendritic branches (A), synaptophysin-positive synaptic boutons (B) and neurofilament-positive nerve fibers (C) in the L4-L5 segments of the spinal cord 4 weeks after ventral root avulsion (VRA) or axotomy (AXO) on the injured (inj.) and uninjured side (uninj.). Error bars show S.E.M. P<0.05 is indicated by *, p<0.01 is indicated by ** and p<0.001 is indicated by ***.

Axotomy induced a decrease in the area occupied by MAP2 positive dendrites on the injured side compared to the non-injured side (p<0.01). MAP2 antibody staining occupied 19±1.2% of the ventral horn on the uninjured side, and 15±0.6% of the ventral horn on the injured side (Fig 2A). Evaluation of the ventral horn after ventral root avulsion showed a significant and strong decrease in the presence of MAP2 positive dendrites on the injured side compared to the uninjured side (p<0.001). MAP2 staining occupied 23±1.4% of the ventral horn of the uninjured side, while MAP2 occupied 4.8±0.5% on the side of injury, indicating close to a five-fold decrease in dendrites (Fig 2A).

Analysis of the density of synapses after ventral root avulsion showed a significant decrease in the density of synaptophysin positive synapses in the ventral horn of the injured side compared to the non-injured side (p<0.001), with the staining covering 3.3±0.2% of the ventral horn area of the former and 7.1±0.6% of the latter (Fig 2B). Following axotomy no significant difference in the density of synaptophysin positive synapses was observed (p>0.05) (Fig 2B).

Following axotomy, the analysis of density of pan-neurofilament positive axons in the ventral horn showed no statistically significant difference between the axotomised and non-injured side (Fig 2C). In animals subjected to ventral root avulsion, a significant decrease of pan-neurofilament positive axons was found on the injured side compared to the uninjured side (p<0.001), with the staining covering 1.2±0.2% of the ventral horn of the former and 5.5±0.5% of the latter (Fig 2C).

We also compared the ventral horn on the uninjured side after ventral root avulsion and axotomy. We found that ventral root avulsion caused an increase in the density of dendrites (p<0.05) and axons (p<0.05) compared with axotomy (Fig 2A and 2C). These findings indicate the onset of a sprouting reaction on the uninjured side following ventral root avulsion, which is not seen after the less severe axotomy injury.

#### The density of microglial and astroglial cells

**Fig 3:**
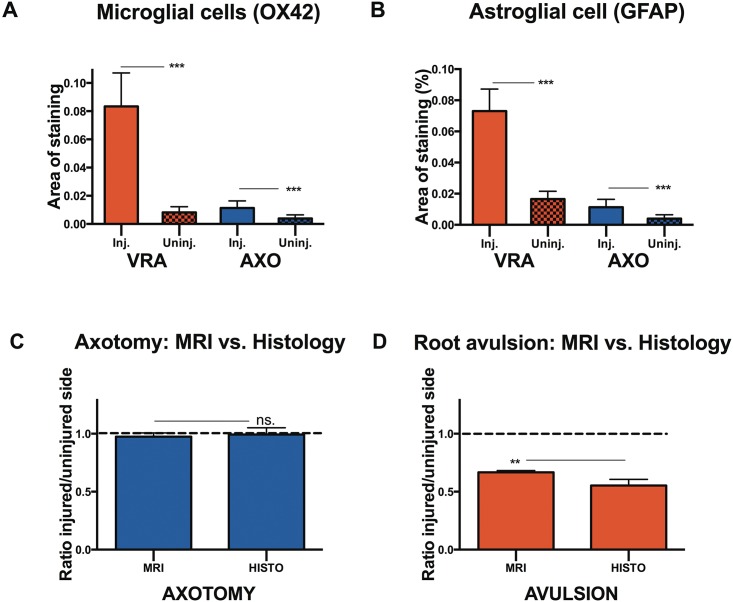
Quantification of the glial response and assessment of correlation between MRI and histological data. Histogram showing the relative tissue area occupied by OX42-positive microglial cells (A) and and GFAP-positive astroglial (B) in the L4-L5 segments of the spinal cord 4 weeks after ventral root avulsion (VRA) or axotomy (AXO) on the injured (Inj.) and uninjured side (Uninj.). Histogram showing the comparison between the relative area ratio following axotomy (C) and ventral root avulsion (D), as measured on MRI images (MRI) and histological preprations stained with NeuN (HISTO). Error bars show S.E.M. p<0.01 is indicated by **, p<0.001 is indicated by *** and ns. indicates lack of statistical significance.

Changes in the presence of microglial cells and astroglial cells was studied by staining with OX42 and GFAP, respectively. Studying the microglial reaction, we found that axotomy induced an almost four-fold increase in the presence of OX42 positive microglia in the ventral horn of the injured side compared to the non-injured side (p<0.01) (Fig 3A). After ventral root avulsion we found a significant (p<0.001), ten-fold, increase in the presence of OX42 microglia in the ventral horn on the side of avulsion compared to the uninjured side (Fig 3A).

Regarding the reaction of astroglial cells after axotomy, we found a significant increase in the presence of GFAP positive astroglial cells on the injured side compared to the uninjured side (p<0.001). Astroglial cells covered 1.7%±0.18% of the ventral horn of the uninjured side and 3.2±0.18% on the injured side (Fig 3B). After ventral root avulsion, the increase in the density of GFAP positive astroglial cells in the ventral horn, compared to the non-injured side, was strong and statistically significant (p<0.001). The density in the injured ventral horn was 7.3±0.4%, compared with 1.7±0.14% for the uninjured side (Fig 3B).

### MRI vs. histology: A comparison

Principally, the same protocol was employed for determining the ventral horn neuron pool size on histological preparations and the ventral horn size as measured in MRI images. We could therefore compare the injured/uninjured area ratio yielded by the two methods after both axotomy and ventral root avulsion. After axotomy, no significant difference in ration was found between the two methods (p>0.05) (Fig 3C). When comparing the area ratio after ventral root avulsion, we found a significant difference (p<0.01), with the ratio calculated from MRI image measurements being higher than calculations based on images of histological preparations (Fig 3D).

## Discussion

This study demonstrated that *in vivo* MRI can be used to differentiate preganglionic avulsion nerve injury and sciatic axotomy. The sciatic nerve injury caused no changes in the ventral horn area as assessed by MRI of the spinal cord segments corresponding to the injured nerves, while the preganglionic avulsion injury induced a severe reduction of the ventral horn area. We further investigated the underlying histological changes, establishing that avulsion caused shrinkage of the ventral horn area, with severe loss of neurons, axons, dendrites and synapses as well as an increased presence of microglial cells and astrocytes. Sciatic axotomy caused no significant shrinkage of the ventral horn area, no significant loss of axons and synapses, and only a mild loss of dendrites. Furthermore, the density of microglial and astroglial cells was only moderately elevated.

It is known from previous studies that ventral root avulsion causes detrimental metabolic, inflammatory and morphological changes to occur in the affected spinal cord segments [17–19], eventually leading to an extensive death of motoneurons. [5] The loss of motoneurons occurring after ventral root avulsion leads to a subsequent loss of dendrites, axons and synapses, disrupting the contact between motoneurons and interneurons. We speculate that this interruption in the ventral horn “neuronal circuitry” may have a detrimental effect on the survival of the general neuron population. Indeed, it is well known that the programmed cell death of motoneurons is paralleled by apoptotic death of interneurons. [20] The general reduction in the number of cells in the ventral horn may subsequently decrease the total size of the neuron pool. Axotomy did not cause a loss of motoneurons, synapses and axons, and only a mild decrease of dendrites 4 weeks after injury, which is generally consistent with previous findings. [15, 21] We speculate that these features may contribute to the sparing of the interneuron population, thus enabling the preservation of the ventral horn morphology and size. The effects on motoneuron and interneuron survival may explain our finding that, while axotomy causes no shrinkage in size of the ventral horn area occupied by neurons, ventral root avulsion causes a severe shrinkage of the ventral horn size after injury.

The presence or absence of changes in ventral horn size detected using MRI after ventral root avulsion and sciatic axotomy respectively may in part be attributed to the direct effects on the survival of ventral horn neurons. However, we also suspect that the severe loss of dendrites and axons might account for a part of the signal decrease seen on MRI image after ventral root avulsion. The proposed effects on dendrites and axons were confirmed when examining the histological material, showing a great decrease in the density of dendrites and axons after ventral root avulsion. In the case of axotomy, the mild decrease in the density of dendrites and no apparent decrease in the density of axons, may account for the absence of changes in MRI images. These findings may be contributing factors to the discrepancy in ventral horn morphology, as seen on MRI pictures, between the two types of injury.

Since the same software and the same basic protocols were used for both assessment of the ventral horn area on MRI-images and assessment of the ventral horn neuron pool, we wanted to further investigate the congruence between the two methods. When comparing the ventral horn shrinkage after ventral root avulsion as measured on MRI images with the shrinkage as measured on histological material, a discrepancy was noted. Measurements of MRI images were revealed to underestimate the loss of ventral horn area after ventral root avulsion when compared with histological preparations. While the mean ratio between the ventral horn area of the injured side and healthy side is 66% as measured using MRI, the corresponding mean ratio for measurements of the neuron pool in histological preparations was 45%. The discrepancy may be secondary to the possibility of the signal detected using MRI being yielded by all cells and structures found in the grey matter, not only motoneurons and interneurons. Indeed, when studying the presence of microglial cells and astrocytes after ventral root avulsion, a ten-fold increase in the former and more than four-fold increase in the latter was observed in the grey matter on the injured side, compared with the non-injured side. We speculate that the activation of microglia and astroglia may account for an increased signal, thus slightly “masking” a general shrinkage in ventral horn size. Despite lack of perfect congruence between the size of the ventral horn nerve pool as measured in histological preparations and the ventral horn as measured with MRI, we have successfully shown that *in vivo* MRI scanning of the spinal cord ventral horn can be used to differentiate pre- and postganglionic nerve injury to the corresponding nerves.

In regard to possible earlier changes in ventral horn size, it has previously been shown that the degeneration of motoneurons is delayed after ventral root avulsion [11]. Our group has also come to a similar conclusion in previous studies, noting degeneration of motoneurons first 8–10 days after ventral root avulsion [13]. The same study found a 30% loss of motoneurons at 2 weeks after injury. Such an extensive loss of motoneurons would most likely produce a decrease in ventral horn size. Therefore, we find it probable that the ventral horn shrinkage may ber detecteble already at two weeks after injury.

We have also previously found that peripheral nerve injury in very young animals induces a considerable loss of sensory neurons in the DRG, with subsequent shrinkage of the ganglion [22]. Therefore, we were interested in possible changes in the dorsal horn size after axotomy. Preliminary results did however not reveal any changes in the dorsal horn size following axotomy.

In clinical practice, spinal CT has long been the “gold standard” in the diagnostics of brachial plexus injuries. Lately MRI, employing high quality myelography sequences, has emerged as an attractive alternative with comparable diagnostic value. [3, 23, 24] The current diagnostic MRI protocols in clinical use are largely based on the imaging of nerve roots, together with the detection of indirect signs of injury. [25, 26] Although constant methodological improvements are being made, due to artifacts and certain interferences, the currently available MRI protocols are yet not considered reliable in the diagnostics of brachial plexus injury. [6]

Our group has previously shown the value of volumetric MRI for the assessment of sensory neuron survival following peripheral nerve injury in rats [22], as well as peripheral nerve injury and brachial plexus injury in humans. [27, 28]. To our knowledge, this study is the first in which the direct effects of preganglionic nerve injury on the ventral horn grey matter have been demonstrated. Furthermore, a successful differentiation of pre- and postganglionic nerve injury was performed as early as 4 weeks after injury. In the clinical setting, an “early repair” is considered to have taken place between 8 and 12 weeks after initial injury [29], with recommended operation of the preganglionic injury within 3 months for an optimal clinical outcome [30]. Furthermore, the diagnostic CT myelography should not be performed earlier than 3–4 weeks after injury, since disruptive blood clots have at this point not yet disappeared and pseudomeningocele, an important indicative sign of avulsion has yet not formed. [6] In this temporal context, a clinical protocol offering the possibility of detecting early secondary signs of preganglionic nerve injury may be very useful. It is our hope that a similar clinical MRI protocol can be developed for future human use. Although not necessarily being a replacement for the current diagnostic protocols, it may prove to be a valuable complementary tool, hopefully aiding early detection of preganglionic injury.

From a pre-clinical scientific point of view, the development of a validated MRI protocol enabling the monitoring of neurodegenerative grey matter changes *in vivo* after preganglionic nerve injury may be very useful. It could provide us with a non-invasive method of monitoring neuroprotective pharmacological treatment. A potent neuroprotective agent capable of salvaging neurons and dendrites and perhaps inducing the sprouting of axons after nerve injury, may be able to alter the grey matter morphology, partially reconstituting the grey matter volume. In these circumstances, a protocol for *in vivo* monitoring of morphological grey matter changes would be ideal, offering the possibility of continuous evaluation of the effects of treatment over time. Moreover, the same subject could be used for studying effects of treatment at different time-points, reducing the number of research subjects needed for experiments. By comparing the results of neuroprotective treatment in the same particular animal over time, interindividual variation may also play less of a role, possibly improving the statistical quality of the results.

In conclusion, this study presents a novel, histologically validated MRI based approach for the differentiation of pre- and postganglionic nerve injury in the rat nerve injury model. The described protocol may serve as a new and non-invasive method of following the effects of neuroprotective treatment. It is also our hope that this may be a starting point for the development of a clinically useful protocol for the differentiation of pre- and postganglionic nerve injury in brachial plexus injury patients.

## Abbreviations

BPI: brachial plexus injury
CT: computer tomography
DICOM: data imaging and communications in medicine
DRG: dorsal root ganglion
MAP2: microtubule associated protein 2
MRI: magnetic resonance imaging
NeuN: feminizing locus on X-3
OX42: Anti-CD11b/c antibody
PBS: Phosphate-buffered saline
PFA: paraformaldehyde
SYN: synaptophysin
TR: repetition time
TurboRARE: turbo rapid acquisition with relaxation enhancement
VH: ventral horn
